# The Histone Methyltransferase SETDB1 Modulates Survival of Spermatogonial Stem/Progenitor Cells Through NADPH Oxidase

**DOI:** 10.3389/fgene.2020.00997

**Published:** 2020-10-02

**Authors:** Xueliang Li, Xiaoxu Chen, Yingdong Liu, Pengfei Zhang, Yi Zheng, Wenxian Zeng

**Affiliations:** Key Laboratory of Animal Genetics, Breeding and Reproduction of Shaanxi Province, College of Animal Science and Technology, Northwest A&F University, Yangling, China

**Keywords:** SETDB1, H3K9me3, NOX4, ROS, spermatogonial stem cell

## Abstract

SETDB1, a histone H3 lysine 9 (H3K9) methyltransferase, is crucial in meiosis and embryo development. This study aimed to investigate whether SETDB1 was associated with spermatogonial stem cells (SSC) homeostasis. We found that knockdown of *Setdb1* impaired cell proliferation, led to an increase in reactive oxygen species (ROS) level through NADPH oxidase, and *Setdb1* deficiency activated ROS downstream signaling pathways, including JNK and p38 MAPK, which possibly contributed to SSC apoptosis. Melatonin scavenged ROS and rescued the phenotype of *Setdb1* KD. In addition, we demonstrated that SETDB1 regulated NADPH oxidase 4 (*Nox4*) and *E2F1*. Therefore, this study uncovers the new roles of SETDB1 in mediating intracellular ROS homeostasis for the survival of SSC.

## Introduction

Male fertility depends on spermatogenesis, by which the haploid spermatozoa generate in the testes. This process starts with the mitosis of the spermatogonial stem cells (SSCs), followed by meiosis of spermatocytes. Finally, the haploid spermatids transform into spermatozoa (Kanatsu-Shinohara and Shinohara, 2013). This highly organized process of spermatogenesis requires timely coordinated gene expression that is regulated at the transcriptional and post-transcription levels (McSwiggin and O’Doherty, 2018). Histone modification has been implicated in the regulation of gene expression.

Histone H3 lysine 9 (H3K9) can be methylated by the methyltransferase SETDB1 (Mozzetta et al., 2015). Notably, the global level of H3K9me3 and SETDB1 gradually increases during development of the testes (An et al., 2014). Loss of *Setdb1* resulted in a reduced number of PGCs and postnatal hypogonadism (Liu et al., 2015). Moreover, depletion of *Setdb1* at postnatal day 7 caused germ cell apoptosis at the pachytene stage and defects in XY body formation (Hirota et al., 2018). *Setdb1* depletion induced SSC apoptosis through upregulating apoptotic inducers and downregulating apoptotic suppressors, and upregulating cytochrome *c* oxidase subunit IV isoform 2 (*Cox4i2*) through decreasing H3K9me3 (An et al., 2014). The up-regulation of COX4i2 is associated with elevated mitochondria-produced reactive oxygen species (ROS) (Singh et al., 2009).

The active NADPH oxidase (NOX) generates superoxide, which spontaneously recombines with other molecules to produce reactive free radicals (Katsuyama et al., 2012; Lambeth and Neish, 2014). Under physiological conditions, the intracellular ROS are thought to act as a second messenger in cell signaling (Bigarella et al., 2014; Lambeth and Neish, 2014; Wang et al., 2018). Recent studies found that ROS generated by NOX1 and NOX3 was essential for SSC self-renewal (Morimoto et al., 2013, 2015). Ablation of *Nox1* severely compromises SSC self-renewal, and *Nox3*-depletion causes apoptosis and impairs SSC proliferation. However, the accumulated ROS is toxic to the cells. Enhancing the expression of NOX4 in cardiac myocytes induces apoptosis and mitochondrial dysfunction (Ago et al., 2010). Excessive ROS causes apoptosis through the p38 MAPK-p16 pathway in hematopoietic stem cells (Ito et al., 2006). High levels of ROS could also induce oxidative stress and activation of FOXO4 that is a regulator of cell cycle, cell death, and cell metabolism (Essers et al., 2004; Urbich et al., 2005; Eijkelenboom and Burgering, 2013). Importantly, oxidative stress is associated with male infertility (Bui et al., 2018). Thus, modest levels of ROS benefits cell proliferation, while accumulated ROS impairs cells. However, the function of SETDB1 in intracellular ROS homeostasis remains elusive. In this study, we revealed that *Setdb1* deficiency caused an increased ROS level via the NOX pathway and induced changes in the cell cycle through the JNK-FOXO4 pathway.

## Results

### Knockdown of *Setdb1* Impairs Proliferation and Induces Apoptosis in Spermatogonial Stem Cells

**FIGURE 1:**
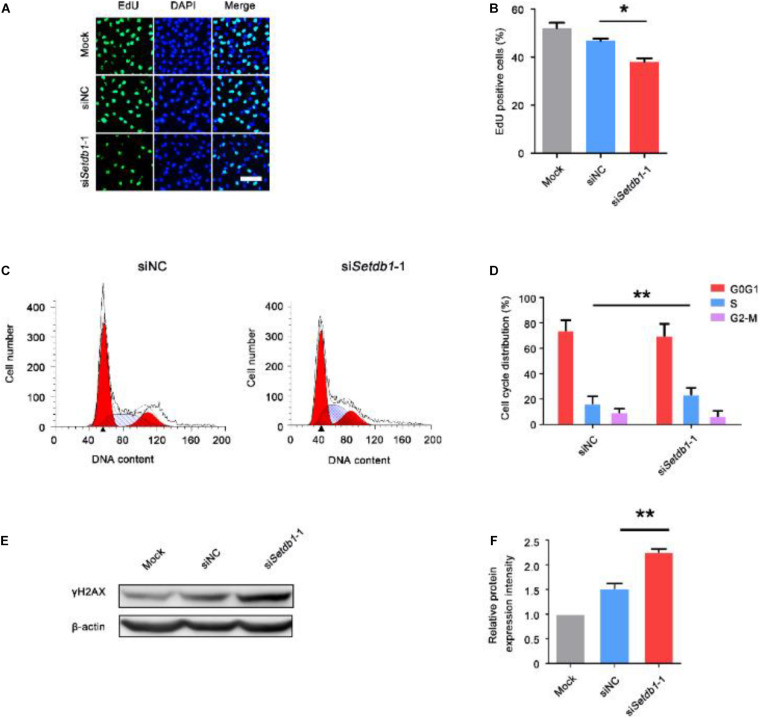
Knockdown of *Setdb1* impairs SSC proliferation. **(A, B)** The EdU incorporation assay showing the proliferation capability of SSCs after transfection with siRNA for 36 h. **(C,D)** Analysis of the cell cycle by flow cytometry after transfection with *Setdb1* siRNA or control siRNA for 36 h **(C)** and the ratios of cells in each phase of the cell cycle **(D)**. **(E,F)** Expression of γH2AX in SSCs shown by Western blot **(E)**. γH2AX intensity analysis was calculated by ImageJ **(F)**. Data are presented as the mean ± SEM of three independent experiments. *P < 0.05, **P < 0.01. Bar = 50 μm.

Using the siRNA oligonucleotides of *Setdb1*, we efficiently downregulated *Setdb1* mRNA expression by approximately 70% (Supplementary Figure S1A). Western blotting analysis confirmed a significant decrease of SETDB1 at protein level after 48 h transfection (Supplementary Figures S1B,C). As shown in Figures 1A,B, the proliferation rate reduced in *Setdb1*-KD cells compared with the control group (Figures 1A,B). Flow cytometry analysis further confirmed that higher ratio of S phase cells in *Setdb1* KD than that of the control at 36 h post transfection (Figures 1C,D). Meanwhile, *Setdb1* depletion induced apoptosis at 48 h post transfection (Supplementary Figures S1D,E). Similar to the previous report, *Setdb1* KD caused an increase of double-strand DNA breaks (Figures 1E,F) (Supplementary Figures S1F,G) (Kim et al., 2016). Interestingly, overexpression of *Setdb1* had no effect on cell survival (Supplementary Figures S1H,J). These observations confirm that SETDB1 is required for the maintenance of SSCs.

### Suppression of *Setdb1* Induces ROS Accumulation and NOX Expression

**FIGURE 2:**
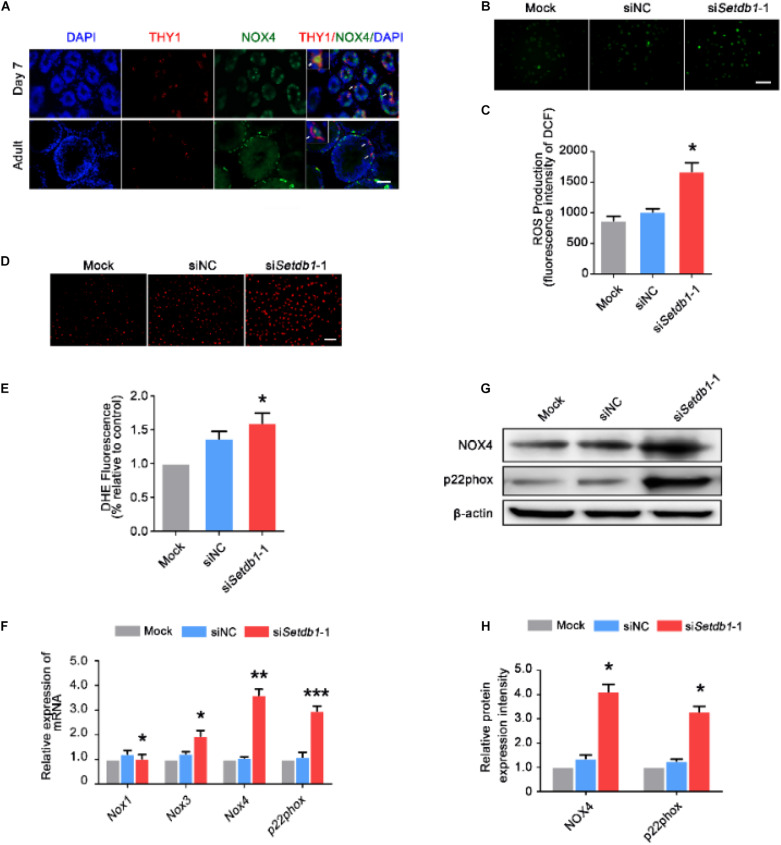
Suppression of SETDB1 induces ROS accumulation and the expression of NADPH oxidase. **(A)** Immunohistochemical analysis of NOX4 and THY1 (an SSC marker) in the testes. **(B,C)** Representative images of DCFH-DA evaluation for ROS production **(B)**. DCFH-DA method was used to analyze ROS level after *Setdb1* knockdown (*n* = 3) **(C)**. **(D,E)** Representative images of dihydroethidium fluorescence staining that evaluation for ROS production **(D)**. Quantitative analysis of DHE relative intensity (*n* = 3) **(E)**. **(F)** The mRNA expression of *Nox1*, *Nox3*, *Nox4*, and *p22phox* upon *Setdb1* knockdown. **(G)** The protein expression of NOX4 and p22phox measured by Western blot analysis. β-actin is used as loading control. **(H)** The protein expression of NOX4 and p22phox were quantitative by ImageJ. Data are presented as the mean ± SEM of three independent experiments. *P < 0.05. Bar = 100 μm.

To clarify the expression of NOX4 in male germ cells, we carried out double-immunohistochemistry staining of testes tissue from 7-day-old and adult mice. NOX4 was co-localized with THY1 (SSC/undifferentiated spermatogonia marker) (Figure 2A). Intracellular ROS levels were detected by DCFH-DA and DHE staining. *Setdb1* KD increased the level of total intracellular ROS (Figures 2B,E; Supplementary Figure S2). To test the potential roles of SETDB1 in mediating intracellular ROS homeostasis through NADPH oxidase, we detected NOX expression. *Setdb1* KD caused an increase of expression of *Nox3*, *Nox4*, and *p22phox* (NOX4 regulatory subunits) (Figure 2F). Western blot assay showed that the level of total NOX4 and p22phox were upregulated (Figure 2G,H). These data suggest that *Setdb1* KD resulted in ROS accumulation possibly by NOX expression in SSCs.

### SETDB1 Activates *Nox4* Expression via Regulating E2F Transcription Factor 1 (E2F1)

**FIGURE 3:**
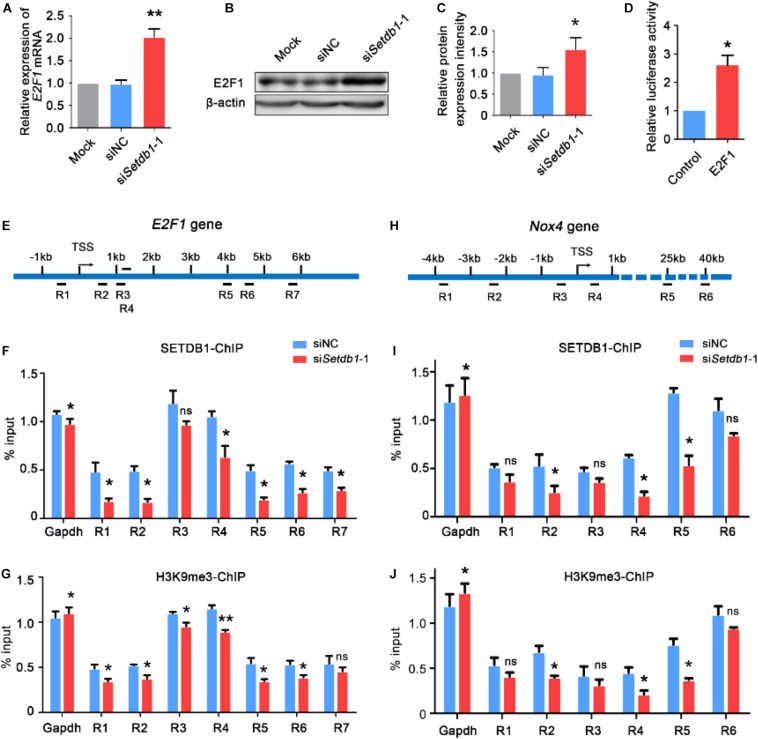
*Setdb1* deficiency in SSCs reduces the enrichment of H3K9me3 at the *E2F1* and *Nox4* transcriptional start site and increases *E2F1* and *Nox4* expression. **(A)** RT-qPCR was performed for detecting *E2F1* expression. **(B,C)** Western blot analysis of *E2F1* gene expression after transfection with *Setdb1* siRNA **(B)**. The protein expression of E2F1 were quantitative by ImageJ **(C)**. **(D)** The luciferase assay showing the activity of *Nox4* promoter fragment in HEK 293T cells. **(E)** Scheme of the *E2F1* promoter used to analyze the enrichment of SETDB1 on different loci (R1–R7) of *E2F1* genomic regions (R1: –549 –329 bp, R2: 496 716 bp, R3: 1167 1278 bp, R4: 1607 1714 bp, R5: 3997 4233 bp, R6: 4548 4707 bp, R7: 5838 5964 bp). **(F,G)** ChIP assays were carried out using anti-SETDB1 **(F)** and anti-H3K9me3 **(G)** antibodies with cell extracts after transfection with *Setdb1* siRNA or control siRNA. **(H)** Scheme of the six different positions (R1–R6) of ChIP primers used to detect the enrichment of SETDB1 and H3K9me3 on *Nox4* genomic regions. (R1: –3850 –3637 bp, R2: –2220 –2044 bp, R3: –431 –593 bp, R4: 513 667 bp, R5: 24861 24987 bp, R6: 40409 40512 bp). **(I,J)** ChIP assays were carried out using anti-SETDB1 **(I)** and anti-H3K9me3 **(J)** antibodies, followed by qPCR based on DNA samples. Data are presented as the mean ± SEM of three independent experiments. Ns, not significant. *P < 0.05.

Western blotting and RT-qPCR assay showed that knockdown of *Setdb1* led to an increase of *E2F1* expression at both mRNA and protein levels (Figures 3A,C). In order to determine whether E2F1 regulates the activity of *Nox4* promoter, the vector of luciferase containing *Nox4* promotor were co-transfected with *E2F1* overexpression vector or empty vector control. As shown in Figure 3D, luciferase reporter assay showed that *E2F1* overexpression significantly increased the luciferase activity compared with that in the empty control group. Hence, E2F1 modulated the activity of *Nox4* promoter (Figure 3D). Since *Setdb1* KD caused the upregulation of E2F1 and NOX4, we test whether *E2F1* and *Nox4* were repressed by SETDB1-mediated histone modification at their promoter region through H3K9me3 (Supplementary Figure S3). Chromatin immunoprecipitation (ChIP) followed by a quantitative real-time PCR (ChIP-qPCR) assay was performed to exam the tentative binding sites of SETDB1 in the promoters of *E2F1* and *Nox4* (Figures 3E,H). We found that the enrichment of SETDB1 and H3K9me3 in the *E2F1* promoter region were only 0.3–1.3% (Figures 3F,G) at these loci. ChIP-qPCR analysis confirmed that there is little enrichment of SETDB1 and H3K9me in the *Nox4* promoter region (Figure 3I,J), suggesting that the regulation of SETDB1 on *Nox4* expression is independent of H3K9me3.

### SETDB1 Regulates Intracellular ROS Homeostasis Through NOX4

**FIGURE 4:**
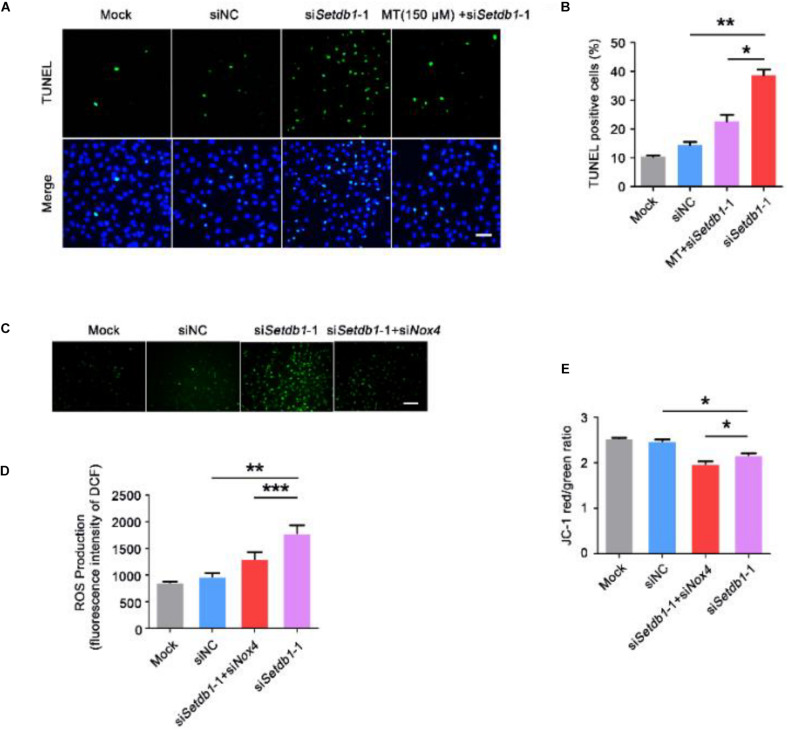
Elimination of ROS helps to prevent the cell death induced by *Setdb1* knockdown. **(A,B)** The TUNEL assay revealing apoptotic SSCs pretreated with ROS scavenger melatonin (MT) before transfection with *Setdb1* siRNA or control siRNA. **(C,D)** Representative images of DCFH-DA evaluation for ROS production **(C)**. DCFH-DA staining showing the generation of ROS after transfection with siRNA for 36 h **(D)**. **(E)** Effects of *Setdb1* knockdown on mitochondrial membrane potential. Data are presented as the mean ± SEM of three independent experiments. *P < 0.05, **P < 0.01. Bar = 50 μm.

To clarify whether *Setdb1*-KD induces apoptosis via the ROS pathway, we pretreated the cells with melatonin, a ROS scavenger, before *Setdb1* knockdown (Tan et al., 2002; Schaefer et al., 2019). As shown in Figures 4A,B, addition of melatonin alleviated the apoptosis induced by *Setdb1* KD (Figures 4A,B), confirming that *Setdb1* KD induces apoptosis through the ROS pathway. We found that addition of melatonin reduced the expressions of *Nox2* and *Nox4* in SSCs, which are similar to preciously published results (Supplementary Figure S4; Najafi et al., 2019). These results indicate that abolition of ROS partially rescued the death phenotype.

**FIGURE 5:**
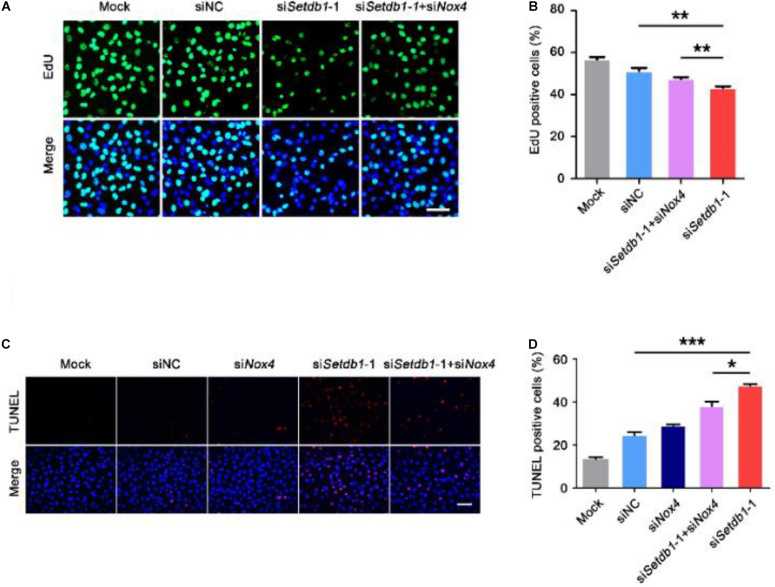
SETDB1 regulates intracellular ROS homeostasis through NADPH oxidase 4. **(A,B)** The EdU incorporation assay showing the proliferation capability of SSCs. **(C,D)** The TUNEL assay showing apoptotic SSCs. Data are presented as the mean ± SEM of three independent experiments. *P < 0.05, **P < 0.01, ***P < 0.005. Bar = 100 μm.

To test whether the ROS level is upregulated by NOX4, we co-transfected specific siRNA against *Setdb1* and *Nox4* and analyzed the knockdown efficiency (Supplementary Figure S5). As shown in Figures 4C,D, ROS were decreased in cells co-transfected with both siRNAs against *Setdb1* and *Nox4* compared with the group that was solely transfected with the *Setdb1* siRNA (Figures 4C,D). To address whether *Setdb1* KD led to mitochondrial dysfunction by upregulating NOX4, both siRNAs of *Setdb1* and *Nox4* were introduced to the cells simultaneously. JC-1 assay showed that mitochondrial dysfunction was reduced in cells co-transfected with both siRNAs of *Setdb1* and *Nox4* compared with the group only transfected with the *Setdb1* siRNA (Figure 4E). Subsequently, we detected the role of NOX4 in apoptosis and cell proliferation. As shown in Figures 5A,B, *Nox4* KD could partly alleviate the phenotype induced by *Setdb1* KD (Figures 5A,B). In addition, TUNEL positive cells were reduced in cells co-transfected with both *Setdb1* siRNA and *Nox4* siRNA compared with the group transfected with the *Setdb1* siRNA (Figures 5C,D). Based on these data, we conclude SETDB1 regulates intracellular ROS homeostasis through NOX4.

### *Setdb1* Knockdown Activates p38/JNK-FOXO4 Pathway

**FIGURE 6:**
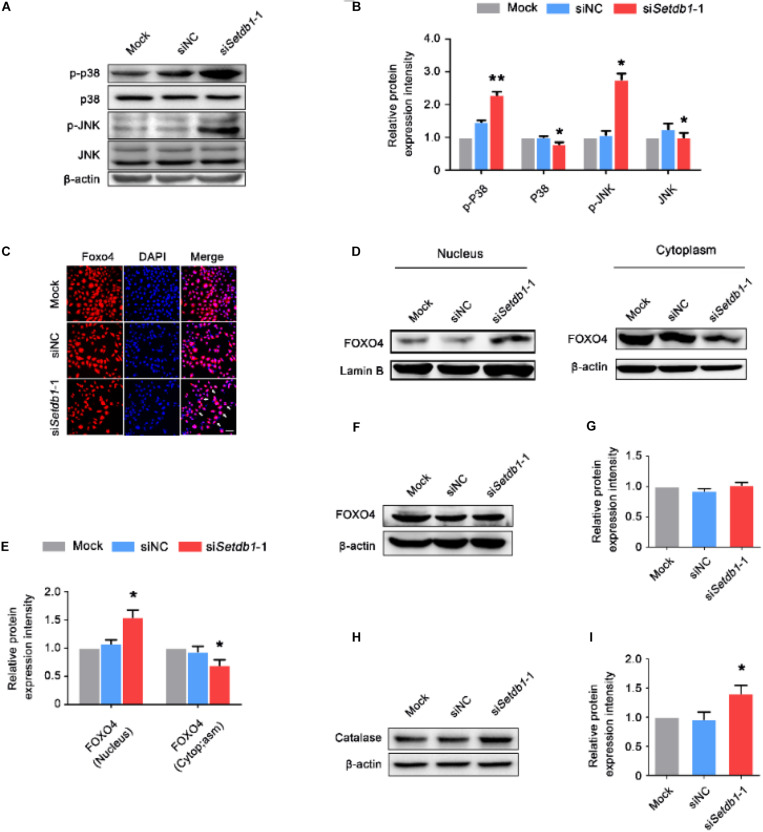
*Setdb1* knockdown activates p38/JNK-FOXO4 pathway. **(A, B)** Western blot analysis showing the phosphorylation of p38 MAPK and JNK after *Setdb1* depletion **(A)**. The expression of p-P38, P38, p-JNK, and JNK proteins were calculated by Image J **(B)**. **(C)** Immunofluorescence staining of FOXO4 in SSCs. FOXO4: red, DAPI: blue. **(D, E)** Immunoblotting for FOXO4 in cytoplasm and nuclei after downregulation of *Setdb1* by specific siRNA **(D)**. Intensity analysis of FOXO4 expression in the cell components was quantitative by ImageJ **(E)**. β-actin and Laminin B are used as loading controls. **(F, G)** Western blot analysis for FOXO4 after transfection with *Setdb1* siRNA for 48 h **(F)**. Quantitative result was illustrated for FOXO4 **(G)**. **(H, I)** Western blot analysis of Catalase in SSCs transfected with *Setdb1* siRNA for 48 h **(H)**. The protein expression of Catalase was calculated by ImageJ **(I)**. Data are presented as the mean ± SEM of three independent experiments. *P < 0.05, **P < 0.01. Bar = 100 μm.

We examined whether SETDB1 mediated the phosphorylation of p38 MAPK and c-jun N-terminal kinase (JNK). We found that *Setdb1* KD led to the activation of p38 and JNK signaling in SSCs (Figures 6A,B).

We further investigated whether *Setdb1*-KD induced activation and translocation of FOXO4. The immunofluorescence assay showed *Setdb1* KD resulted in FOXO4 translocation (Figure 6C). To further confirm the nuclear translocation of FOXO4 after *Setdb1* KD, we extracted protein of the nucleus and cytoplasm. The western blot assay confirmed that FOXO4 translocated from the cytoplasm to the nucleus (Figures 6D, E). However, the expression of FOXO4 almost did not change at 48 h after *Setdb1* KD (Figures 6F,G). *Setdb1* KD upregulated the expression of the target gene encoded Catalase (Figures 6H,I). These results suggest that *Setdb1* KD activates the p38/JNK-FOXO4 pathway.

## Discussion

SETDB1 catalyzes H3K9me3, which is a repressive marker (Mozzetta et al., 2015). In the present study, we found that SETDB1 repressed expression of *Nox4* and *E2F1* and mediated ROS levels.

NOX consumes oxygen to generate O_2_^–^ using NADPH as an electron donor, and the O_2_^–^ subsequently forms H_2_O_2_ (Katsuyama et al., 2012; Bigarella et al., 2014). Previous studies have shown that ROS generated by NOX enhanced growth factor signaling and acts as anti-microbial molecules (Nathan and Cunningham-Bussel, 2013). Excessive ROS production induces cellular injury and lipid peroxidation (Su et al., 2019). In this study, we found that *Setdb1* KD induced accumulation of ROS and upregulation of *Nox3*, *Nox4*, *p22phox*, and *E2F1*. Importantly, melatonin alleviated the apoptosis in *Setdb1*-KD group. Co-transfecting with siRNAs of *Nox4* and *Setdb1* simultaneously resulted in the decrease of ROS and increase of mitochondrial membrane potential compared with the *Setdb1* depleted cells. Furthermore, melatonin reduced the expression of *Nox2* and *Nox4*, which is consistent with the previous report (Najafi et al., 2019). Therefore, melatonin alleviated *Setdb1*-KD induced SSC apoptosis, probably by down-regulating *Nox2* and *Nox4*. The excess ROS was generated from NOX4 and was responsible for the apoptosis in *Setdb1*-KD cells. In this study, we also found that *Setdb1* KD led to increase of *Nox3*. Together, SETDB1 mediates ROS homeostasis and likely keeps ROS below a threshold level via NADPH oxidase.

It has been reported that ROS generated by NOX4 was associated with DNA damage (Weyemi et al., 2012), which was consistent with the present findings on SSCs. Except for double-strand DNA breaks, ROS activates the signal-transducing molecules including JNK, p38, and FOXO4 (Ma et al., 2002; Essers et al., 2004; Hua et al., 2017; Zheng et al., 2017). In mammals, the FOXO family consists of four members (FOXO1, FOXO3, FOXO4, and FOXO6) (Eijkelenboom and Burgering, 2013). These FOXO transcription factors regulate multiple cellular pathways, including apoptosis, inflammation, proliferation, oxidative stress resistance, and aging (Henderson and Johnson, 2001; Lin et al., 2001; Tuteja and Kaestner, 2007a, b; Zanella et al., 2010; Genin et al., 2014; Webb and Brunet, 2014; Murtaza et al., 2017; Jiramongkol and Lam, 2020). Meanwhile, FOXO nuclear translocation triggers apoptosis by inducing the expression of death genes, such as the *FasL*, and thereby participates actively in the process of apoptosis (Brunet et al., 2004). In this study, knockdown of *Setdb1* activated the ROS-JNK signaling pathway and FOXO4 that was translocated into the nuclei, which led to an increase of expression of the *Catalase* gene (FOXO4 target gene) that encodes an anti-oxidant enzyme (Nandi et al., 2019). Taken together, we propose that *Setdb1* KD activates ROS downstream signaling pathways, which partially contributes to the apoptotic phenotype in SSCs.

SETDB1 is involved in heterochromatin formation and transcription silencing via histone H3 methyltransferase activity (Zhu et al., 2020). In this study, we found that *Setdb1* KD led to the upregulation of *Nox4* and *E2F1*. ChIP-qPCR showed that 0.3–1.4% of input for SETDB1 and H3K9me3 at the loci of the *E2F1* and *Nox4* promoters, indicating that SETDB1 does not target *E2F1* and *Nox4* promoters. Recent studies revealed that *Setdb1* KD resulted in the activation of endogenous retroviruses (ERVs) and the long terminal repeat (LTR) and led to dysregulation expression of neighboring genes (Tan et al., 2012). Thus, SETDB1 may regulate the expression of *Nox4* and *E2F1* due to silencing of cis-regulatory elements or retrotransposons in SSCs.

NOX4 was involved in various physiological processes such as apoptosis and differentiation in various cell types (Pedruzzi et al., 2004; McKallip et al., 2006; Carmona-Cuenca et al., 2008; Caja et al., 2009). It was reported that SETDB1 was recruited to the *E2F1* promoter and co-operated with Alien complex to regulate the expression of *E2F1* (Hong et al., 2011). Meanwhile, E2F1 positively regulates the transcription of *Nox4* in vascular smooth muscle cells (Zhang et al., 2008). In this study, the expression of E2F1 and NOX4 were elevated in *Setdb1*-KD group. The expression of E2F1 was upregulated in *Setdb1*-KD cells, which in turn lead to upregulation of NOX4.

The role of SETDB1 has been explored extensively in the development of male germ lines (An et al., 2014; Liu et al., 2015, 2017; Hirota et al., 2018; Mochizuki et al., 2018). SETDB1 is recruited to repress ERVs transcription via H3K9me3 in primordial germ cells (Liu et al., 2015), and suppresses the expression of *Dppa2*, *Otx2*, and *Utf1* during PGC determination (Mochizuki et al., 2018). *Setdb1* knockout disrupts spermatogenesis and expression of meiosis-related genes (Hirota et al., 2018). Therefore, SETDB1 regulates different clusters of genes in the development of male germ cells. It would be interesting to further elucidate the mechanisms of recruitment in SETDB1 to different genes.

In conclusion, SETDB1 regulates the expression of *E2F1* and *Nox4*. *Setdb1* depletion causes the derepression of *E2F1* and upregulation of *Nox4*. On the other hand, NOX4 was upregulated by E2F1 dysregulation. Thus, NOX4 contributes to ROS generation and activates ROS downstream signaling pathways. Meanwhile, excessive amounts of ROS induces cell cycle arrest and apoptosis in SSCs. This study will provide a new perspective on SETDB1 function and understanding of male infertility.

## Materials and Methods

### Cell Culture and Transfection

C18-4 cell line was obtained from Dr. Zuping He at Shanghai Jiao Tong University, China. The cell line was established from mouse type A spermatogonia from 6-day-old mice (Hofmann et al., 2005). C18-4 cells were maintained in Dulbecco modified Eagle medium (DMEM)/F12 (Hyclone, Logan, UT, United States) supplemented with 10% fetal bovine serum (BI, Israel), 100 U/ml penicillin and streptomycin (Gibco), 100 mM non-essential amino acids (Gibco), and 2 mM L-glutamine (Gibco) at 37°C and 5% CO2. The 293T cell line was cultured in DMEM/Basic medium supplemented with 10% fetal bovine serum, 100 U/ml penicillin and streptomycin, 100 mM non-essential amino acids, and 2 mM L-glutamine at 37°C and 5% CO2.

A pair of *Setdb1* small interfering RNAs (siRNAs), *Setdb1*-1 and *Setdb1*-2, were ordered from GenePharma (Shanghai, China). Sequences of mouse *Setdb1* siRNA were as follows: 5′-CCAACC UGUUUGUCCAGAAUGUGUU-3′ (*Setdb1*-1), 5′-UCAAGUUUGGCAUCAAUGAUGUAGC-3′ (*Setdb1*-2), 5′-UU CUCCGAACGU GUCACGUTT-3′ (Scramble). The sequence of mouse *Nox4* siRNA was as follows: 5′-GTAGGAGAC TGGACAGAAC-3′. The cells were transfected with siRNAs using Lipofectamine 2000 Transfection Reagent (Invitrogen) according to the manufacturer’s instructions.

### Reverse Transcription-Quantitative Polymerase Chain Reaction (RT-qPCR)

Total RNA was extracted using RNAiso Plus reagent (TaKaRa, Dalian, China). RT-qPCR was performed as described previously using the primers listed in Supplementary Table S1 (Chen et al., 2017).

### Western Blot

The cells were transfected with *Setdb1* siRNA for 48 h. Approximately 30 μg protein was separated by 8–12% SDS-PAGE and transferred to PVDF membranes (Millipore). The membranes were probed using the following primary antibodies: NOX4 (1:500; NB110-58849; Novus), beta-Actin (1:2000; CW0096; CWBIO), SETDB1 (1:1000; 11231-1-AP; Proteintech), E2F1 (1:500; sc-193; Santa Cruz Biotechnology), FOXO4 (1:500; sc-5221; Santa Cruz Biotechnology), Lamin B (1:500; sc-6217; Santa Cruz Biotechnology), JNK (1:500; sc-7345; Santa Cruz Biotechnology), p-JNK (1:400; WL01813; WanleiBio), γH2AX (1:000; 2577; Cell signaling technology), p38 (1:500; sc-7972; Santa Cruz Biotechnology), and p-P38 (1:500; sc-17852-R; Santa Cruz Biotechnology). All were used as the manufacturer’s recommendation. The secondary antibodies were horse radish peroxidase-linked anti-mouse, anti-rabbit, or anti-goat IgG for 2 h at room temperature. The membranes were visualized on a Bio-Rad Chemidoc XRS using a Western Bright ECL Kit (Bio-Rad, Berkeley, CA, United States).

### ROS Measurement

Intracellular ROS was determined using the 2′, 7′-dichlorofluorescein diacetate (DCFH-DA, Beyotime) and Dihydroethidium (DHE, Beyotime) according to the manufacturer’s instructions. Cells were incubated with 10 μM 2′, 7′-dichlorofluorescein diacetate or dihydroethidium at 37°C for 30 min. Subsequently, the fluorescence signals of the cells were observed using a multi-detection microplate reader. The excitation/emission of DCFH-DA is 488/525 nm, and the excitation/emission of DHE staining are 370/420 and 300/610 nm.

### Cell Cycle Assay

The cell cycle analysis was performed with Flow cytometry. The cells were harvested at 36 h post transfection of *Setdb1* siRNA or control siRNA. After being fixed in 70% cold ethanol, the cells were incubated with RNase and finally stained with 4′,6-diamidino-2-phenylindole (DAPI, Bioworld). DNA content was analyzed by Flow cytometry (BD FACSAria^TM^ III, United States). The data were analyzed with ModFit LT 5.0.

### TUNEL Staining

Apoptotic cells were detected with TUNEL BrightGreen or BrightRed Apoptosis Detection Kit (Vazyme, Nanjing, China) according to the manufacturer’s instructions. The cells were seeded on 96-well plates and transfected with siRNA. After washing with PBS, the cells were fixed in 4% paraformaldehyde (PFA) for 30 min. Then the cells were treated with proteinase K (20 mg/ml) for 5 min at room temperature and incubated in TUNEL reaction mixture at 37°C for 1 h in darkness. The nuclei were counterstained with DAPI (Bioworld). The cells were observed under a fluorescence microscope (Nikon, Tokyo, Japan).

### Immunocytochemistry

The cells were seeded onto a 96-well plate and transfected with siRNA for 48 h. The cells were fixed with 4% PFA for 30 min, permeabilized in 0.5% TritonX-100 for 10 min, and blocked in 3% BSA for 2 h. The cells were incubated with primary antibody for FOXO4 (sc-5221; Santa Cruz Biotechnology) and γH2AX (2577; Cell signaling technology) overnight at 4°C. After washing with PBS, the cells were incubated for 1 h with secondary antibody, followed by incubation with DAPI.

### Immunohistochemistry

Testes from 6-d- and 3-m-old C57BL/6J mice were used for histologic analyses. In brief, the slides (5 μm thick) were blocked with 10% donkey serum for 2 h to block non-specific reactions. The following primary antibodies were used: anti-NOX4 (NB110-58849; Novus) and anti-THY1 (sc-9163, Santa Cruz Biotechnology). The following secondary antibodies were used: Alexa 594-conjugated donkey anti-mouse IgG and Alexa 488-conjugated donkey anti-rabbit IgG (1: 400, Invitrogen). Photomicrographs were captured under a Nikon i90 microscope (Nikon, Tokyo, Japan).

### Plasmid Construction

The 2 kb region of the *Nox4* gene promoter was amplified by polymerase chain reaction (PCR). Subsequently, PCR product was purified using AxyPrepTM PCR Clean-Up Kit (Axygen, CA, United States). The resulting fragments digested by *Kpn*I/BgI II were inserted into the *Kpn*I/BgI II restriction sites of digested pGL3-Basic vector. The ligated mixtures were transformed into competent cells of Escherichia Coli DH5α using the heat shock method.

### Transfections and Luciferase Assays

The 293T cells were transiently transfected using TurbofectTM (Thermo Fisher Scientific) reagent according to the manufacturer’s protocol. The cells were seeded onto 24-well plates, and transfected with 1 μg total plasmid containing 0.5 μg pGL3-basic-*Nox4*, 0.5 μg pCDNA3.1-*E2F1*, and 0.2 μg pRL-CMV, which were transfected as reference plasmid. The transfected cells were cultured for 48 h and analyzed using a dual-luciferase reporter assay system kit (Promega, Madison, WI, United States) according to the manufacturer’s protocol.

### Chromatin Immunoprecipitation-qPCR

Chromatin Immunoprecipitation analysis was performed as previously described using EZ-Magna ChIP A/G (Millipore) (Liu et al., 2017). In brief, the cells were fixed with 1% formaldehyde and lysed in lysis buffer. After the sonication the cell lysates were immunoprecipitated with SETDB1 (11231-1-AP; Proteintech), H3K9me3 (07-442, millipore), or normal IgG (millipore) antibodies. IgG is as a background of the IP. The purified DNA was analyzed by RT-qPCR. The primer was designed by published H3K9me3 ChIP-seq data in mouse undifferentiated spermatogonia cells (Liu et al., 2019). Then, ChIP-qPCR primers were designed around the transcription initiation site, and the size of the product was about 200 bp (Asp, 2018). Finally, the statistical calculation methodology was performed as described previously (Nelson et al., 2006). Briefly, the ChIP-qPCR data output from RT-qPCR software was in the form of Cycle threshold (Ct) values. The relative occupancy of the SETDB1 and H3K9me3 at a locus is measured by the equation 2^(Ctmock-Ctspecific), where Ctmock and Ctspecific are mean threshold cycles of RT-qPCR. Primers were listed in Supplementary Table S2.

### Statistics

The statistical analysis of the differences between two groups was performed by Student’s *t*-test. *P* < 0.05 indicated statistical significance.

## Data Availability Statement

All data used to support the findings of this study are included in the article.

## Author Contributions

XL and XC: conceptualization and writing – original draft preparation. XL, YL, and PZ: methodology. XL: data curation. YZ and WZ: writing – review and editing and funding acquisition. All authors contributed to the article and approved the submitted version.

## Conflict of Interest

The authors declare that the research was conducted in the absence of any commercial or financial relationships that could be construed as a potential conflict of interest.
