# Supplementary material for: The Histone Methyltransferase SETDB1 Modulates Survival of Spermatogonial Stem/Progenitor Cells Through NADPH Oxidase
Source: Front Genet. 2020 Oct 2;11:997. doi: 10.3389/fgene.2020.00997 (PMC7567028; doi:10.3389/fgene.2020.00997)
Supplement: Supplementary file 1 [file Data_Sheet_1.docx]

Supplementary Material

# Table S1. Primers used in qRT-PCR experiments

| gene | Forward Primer (5’ to 3’) | Reverse Primer (5’ to 3’) |
| --- | --- | --- |
| *Setdb1* | ATCCCATTTGCCGACCACTAA | ACCATTGGGCGGTTTGGATAG |
| *Nox1* | AGTGATGTATGCAGCATTTACCA | CCATAGCTGACGTTACCATGAGA |
| *Nox3* | TGGCAGTAAACGCCTATCTGT | CGGAACCCAGAATAACTCGTGTA |
| *Nox4* | CCTTTTACCTATGTGCCGGAC | CATGTGATGTGTAGAGTCTTGCT |
| *p22phox* | TGGACTCCCATTGAGCCTAAA | CTCGGCTTCTTTCGGACCTC |
| *E2F1* | TGCAGAAACGGCGCATCTAT | CCGCTTACCAATCCCCACC |
| *βactin* | GTGACGTTGACATCCGTAAAGA | GCCGGACTCATCGTACTCC |
| *GAPDH* | TGGATTTGGACGCATTGGTC | TTTGCACTGGTACGTGTTGAT |

# Table S2. List of primers used in ChIP experiments

| Position | Forward Primer (5’ to 3’) | Reverse Primer (5’ to 3’) |
| --- | --- | --- |
| *Nox4*-R1 | TGACCATCGTGGCACAGAC | TTGCCGAACACTCCATTTT |
| *Nox4*-R2 | GGCAGACAATCATTAACAGT | CAAGGGTAGCTTGTAGAAAG |
| *Nox4*-R3 | ATAGGAGGAGCAAGTTCAG | GAGACTATCCAAAGCCAGA |
| *Nox4*-R4 | AAAGTCACTGAACACCGATTA | GAAGCTCAGATTCCCTCTA |
| *Nox4*-R5 | TCACTGGACGCACAAAGAC | CCTTGCTATGTTACGGTTG |
| *Nox4*-R6 | CTCTACTGAGCACTAACCCTG | ATTGGAGCAAGCATAAACA |
| *E2F1*-R1 | TTCTGGGTGACAGGATAGGG | CTGGCGAAGCGAACAAACT |
| *E2F1*-R2 | CGGAGCGTCGGAACCATTA | GCAAGCCAGCAGACATCAGT |
| *E2F1*-R3 | CTAAGCAAGTAGCTCAAGACCA | TCCACCCCTACAATGAAGAT |
| *E2F1*-R4 | AAGAGCCAGGCTTAGAGTAGAAA | AAGCACTCACAGCGACCAG |
| *E2F1*-R5 | AGGCAGAGTGATGTAGAGGGAG | TTCACAGTATTGGGAAGAAGGAC |
| *E2F1*-R6 | AGCCTGAGATGGAATCCTGTT | TGTCAAGTAGCCCTAAAGATAAGC |
| *E2F1*-R7 | CAGCCAGACCAAGCCAAAT | AACAGGAGACCCTCAGAAAGG |

# Supplementary Figures


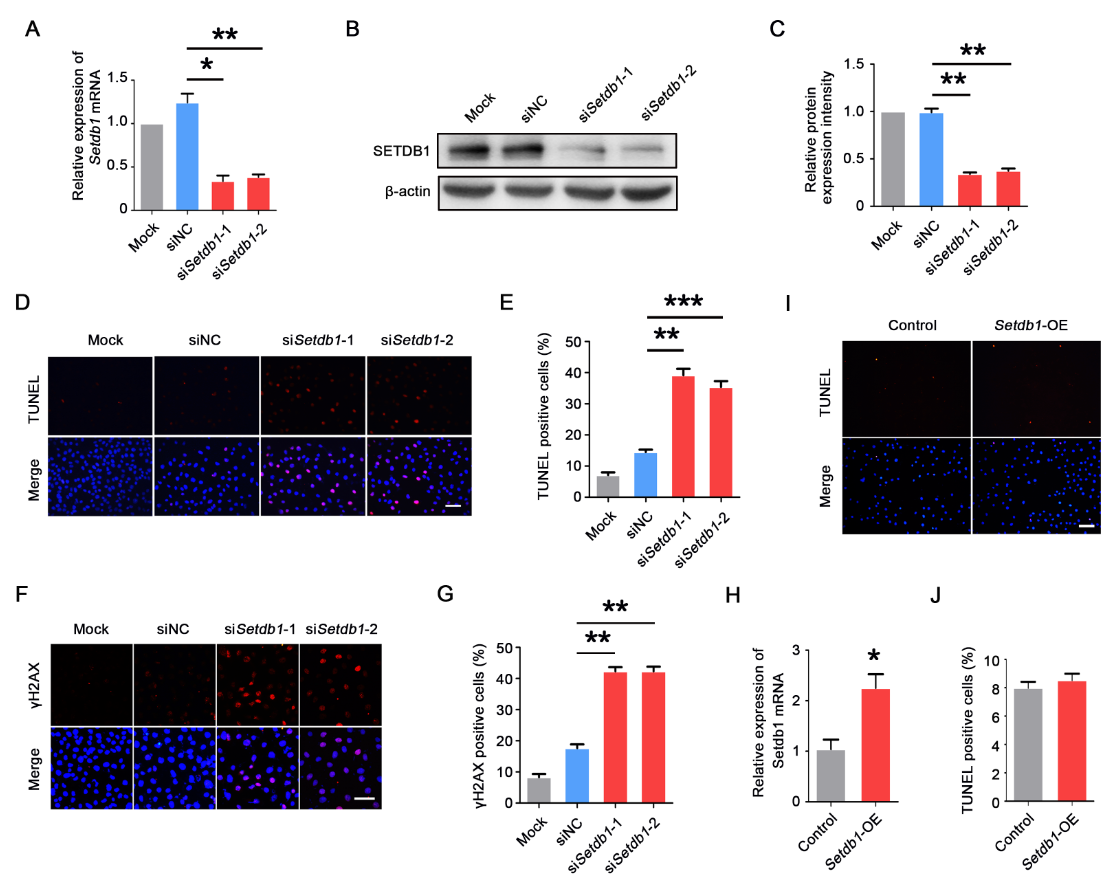


**Supplementary Figure S1.** Effects of *Setdb1*-KD on apoptosis and DNA double-strand breaks in SSCs**. (A-B)** Silence of *Setdb1* expression in SSCs using siRNAs. The *Setdb1* knockdown efficiency was measured by RT-qPCR **(A)** and Western blot analysis **(B)**. β-actin is used as loading control. **(C)** Intensity analysis of SETDB1 was analysed by ImageJ. **(D, E)** SSCs transfected with *Setdb1*-targeting siRNA or control siRNA were subjected to TUNEL staining **(D)**. **(E)** The percentages of TUNEL-positive cells in each group. **(F, G)** Immunocytochemistry of γH2AX in SSC after SETDB1 knockdown **(F)**. Quantification of cells with γH2AX staining **(G)**. **(H)** RT-qPCR results showed the mRNA levels of *Setdb1* in the control and *Setdb1* overexpression samples. **(I, J)** TUNEL assay was performed to analyze apoptotic cells **(I)**. Statistical analysis of the percentage of TUNEL positive cells in each group **(J)**. Data are presented as the mean ± SEM of three independent experiments. ***P*＜0.01, ****P*＜0.005. Bar=100 μm.


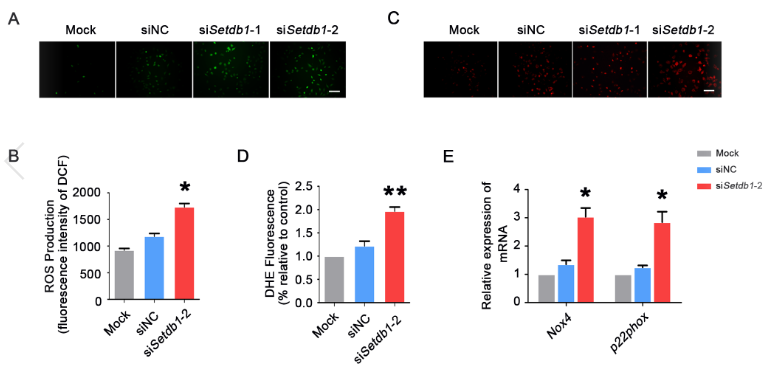


**Supplementary Figure S2.** *Setdb1* KD induced excessive intracellular ROS. **(A, B)** Representative immunofluorescence images of SSCs stained with DCFH-DA **(A)**, quantitative analyses are shown in panels **(B)**. **(C, D)** Intracellular ROS level was detected by fluorescence microscope after DHE staining **(C)**, quantitative analyses are shown in panels **(D)**. **(E)** The mRNA expression of *Nox4* and *p22phox* upon *Setdb1* knockdown. Data are presented as the mean ± SEM of three independent experiments. **P*＜0.05, ***P*＜0.01. Bar=50 μm.


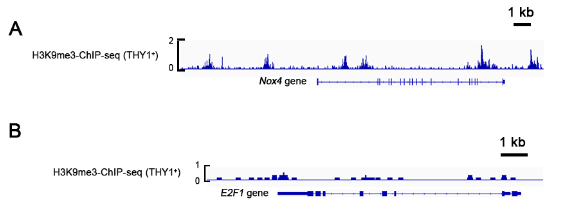


**Supplementary Figure S3**

Genome browser screenshot of the *Nox4* and *E2F1* locus showing the H3K9me3 enrichment in SSCs. **(A)** Genome browser views showing H3K9me3 coverage of the *Nox4*. **(B)** Genome browser views showing H3K9me3 modification of the *E2F1.*


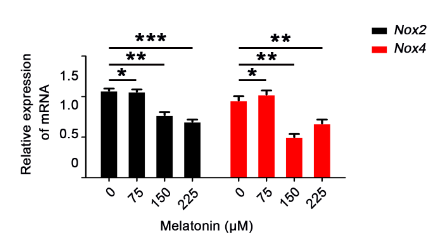


**Supplementary Figure S4** Transcription levels of *Nox2* and *Nox4* were detected after treatment with different does of melatonin (75, 150 and 225 μM) for 48 h in SSCs by RT-qPCR.


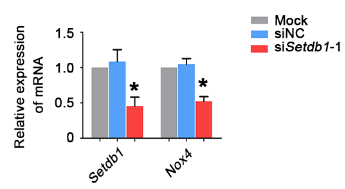


**Supplementary Figure S5.** The *Setdb1* and *Nox4* knockdown efficiency were measured by RT-qPCR analysis after transfection with *Setdb1* and *Nox4* siRNA. Data are presented as the mean ± SEM of three independent experiments. **P*＜0.05.
